# Stents Farmacológicos de Polímero Bioabsorvivel: Uma Presença Sólida nos Laboratórios de Cateterismo, mas será Suficiente?

**DOI:** 10.36660/abc.20260668

**Published:** 2026-11-04

**Authors:** Monica Verdoia, Maria Antonieta Albanez, Andrea Rognoni

**Affiliations:** 1 Nuovo Ospedale degli Infermi Ponderano Itália Nuovo Ospedale degli Infermi, Ponderano – Itália; 2 Real Hospital Português Recife PE Brasil Real Hospital Português, Recife, PE – Brasil

**Keywords:** Stents Liberador de Fármaco com Polímero Bioasorvível, Reestenose, Desfechos clínicos

Várias gerações de stents farmacológicos (SF) surgiram na sequência, buscando reduzir a invasividade das hastes metálicas e as complicações relacionadas ao dispositivo. No entanto, apesar da obtenção de taxas muito baixas de trombose do stent (<1%) e reestenose (<5%), esses eventos ainda impactam os resultados de pacientes com doença arterial coronariana, especialmente em subgrupos anatômicos ou clínicos mais complexos.^1,2^

A redução progressiva da espessura das hastes metálicas, com o desenvolvimento de novos materiais e design de stents, permitiu melhorar a navegabilidade e evitar o aprisionamento excessivo do vaso, sendo mais respeitosa com a vasomotricidade e os ramos colaterais. Além disso, a diminuição do estímulo traumático e inflamatório com hastes mais finas pode explicar a menor ocorrência de reestenose.^3^

De fato, as gerações atuais de SF, variando de 60 a 100 μm, provavelmente atingiram a resolução mínima das hastes, pagando, em contrapartida, o preço da força radial reduzida e de potenciais fraturas, encurtamento e subexpansão, especialmente em lesões calcificadas.^3^

O direcionamento da reestenose com a administração local de moduladores do sistema imunológico, eluídos no lado abluminal do stent, certamente representou o ponto de virada dos stents farmacológicos, com o desenvolvimento da família "-*limus*", que combina os efeitos antiproliferativos e anti-inflamatórios com propriedades de biocompatibilidade. No entanto, os primeiros polímeros utilizados para modular a liberação progressiva desses fármacos na parede do vaso revelaram-se eles próprios pró-inflamatórios e trombogênicos.^1,4^

A terceira geração de stents farmacológicos tentou superar essa limitação introduzindo polímeros biorreabsorvíveis (BP), como o ácido polilático (PLA) e o ácido poli(lático-co-glicólico) (PLGA), que desaparecem completamente quando a eluição do fármaco é concluída, geralmente entre 3 e 12 meses após o implante.

Vários SF de 3ª geração, apresentando pequenas diferenças, conforme mostrado na Tabela 1, foram desenvolvidos, mostrando não inferioridade consistente em relação aos SF de polímero permanente em seus ensaios de validação.^2,5^

**Tabela 1:** Stents farmacológicos atuais de hastes finas e polímero biorreabsorvível

Marca SF	Material das hastes	Espessura das hastes	Medicamento eluído	Tipo de polímero	Duração do polímero	Validação DAPT resumida	Avaliação dos desfechos clínicos	MACE	TLR	ST
Inspiron™	Cromo-cobalto	75 μm	Sirolimus	PLA + PLGA	6 a 9 meses	X	24 meses	6,7%	0,9%	0,6%
Sinergy ™ XD	Platina-cobalto	74 μm	Everolimus	PLGA	3-4 meses	√	12 meses	-	2,6%	0,4%
Orsiro ™	Cromo-cobalto	61 μm	Sirolimus	PLLA	12 a 24 meses	√	60 meses	-	6,3%	0,7%
Biomatrix Alpha ™	Aço inoxidável	112 μm	Biolimus A9	PA	9 meses	X	60 meses	15,7%	9,4% *	2,2%
DESyne BD/BDS ™	Cromo-cobalto	81 μm	Novolimus/ Sirolimus + Rivaroxabana + Argatrobana	PLLA	6 a 9 meses	X	24 meses	-	2,1-4,2%	0-1%
Biomime ™	Cromo-cobalto	65 μm	Sirolimus	PLLA+PLGA	1-2 meses	X	24 meses	7,7%	5,95%	1,19%
Mistent ™	Cromo-cobalto	59 μm	Sirolimus	PLLA+PLGA	1,5 a 2 meses	X	60 meses	15,1%	3,5%	0%
Firehawk ™	Cromo-cobalto	86 μm	Sirolimus	PA	6 a 9 meses	√	60 meses	-	7,6%	2,6%
Combo ™	Aço inoxidável	100 μm	Revestimento luminal de sirolimus + CD34^+^	PA	3 meses	√	12 meses	-	2,5%	0,7%
Supraflex Cruz ™	Cromo-cobalto	60 μm	Sirolimus	PLLA+PLCL+PVP	9 a 12 meses	√	36 meses	-	6,3%	1%
Ultimaster ™	Cromo-cobalto	80 μm	Sirolimus	PLCL	3-4 meses	√	12 meses	-	1,7%	0,7%
Yukon Choice ™	Aço inoxidável	79 μm	Sirolimus	PA	2 a 3 meses	√	120 meses	47,7%	20,3%	2,5%
Nobori™	Aço inoxidável	120 μm	Biolimus A9	PA	9,12 meses	X	60 meses	19,9%	6,3%	0%

* para revascularização do vaso alvo.

SF: stent farmacológico; PLA: ácido polilático; PLGA: ácido polilático-co-glicólico; PA: poliamida; PLCL: poli(L-lactídeo-co-ε-caprolactona); PVP: polivinilpirrolidona; DAPT: terapia antiplaquetária dupla; MACE: eventos cardiovasculares adversos maiores; TLR: revascularização da lesão-alvo; ST: trombose do stent.

No entanto, conforme confirmado por meta-análises recentes,^6,7^ não foi observada redução significativa nos eventos relacionados ao stent com os SF mais recentes, apresentando taxas baixas, mas ainda relevantes, de falha da lesão (FL) e eventos clínicos (7-10% em 1 ano e média de 15% em 5 anos).

O stent Inspiron™ é um stent ultrafino (75 μm) liberador de sirolimus com bifosfonato (BP-sirolimus) (Scitech, Aparecida de Goiânia, Brasil), representando atualmente um dos poucos stents farmacológicos de terceira geração a serem testados em seu primeiro estudo em humanos contra outro stent farmacológico com bifosfonato (BP-SF).^8^ De fato, o estudo DESTINY randomizou 170 pacientes com 194 lesões, na proporção de 2:1, para receber o stent Inspiron™ BP-SES versus o stent Biomatrix BP-BES, mostrando taxas comparáveis de eventos cardíacos adversos maiores combinados (MACE, morte cardíaca, infarto do miocárdio ou revascularização da lesão-alvo) em 5 anos e nenhum caso de trombose do stent.

No presente estudo de Soccol et al.,^9^ o stent Inspiron™ BP-SES foi utilizado em uma coorte muito maior, composta por 2.803 pacientes tratados em 25 centros no Brasil, representando a vida real. Esses pacientes incluíam lesões complexas, em sua maioria do tipo B2 ou C, que necessitavam de mais de um stent, com 5% de reestenose intra-stent (RIS), e subgrupos de alto risco (cerca de 50% com síndrome coronariana aguda e mais de 30% com diabetes mellitus). Apesar disso, a taxa de MACE em 2 anos foi extremamente baixa, de apenas 6,7%, enquanto a FL e a trombose do stent foi semelhante à observada na literatura. Cabe ressaltar que a inclusão de morte cardíaca, em vez de mortalidade por todas as causas, na definição de MACE pode ter contribuído parcialmente para a menor taxa de eventos observada neste estudo.

Na prática, a mortalidade geral ocorreu em cerca de 5% dos pacientes, em consonância com outros registros de grande escala e RCT, e com as conclusões da análise conjunta de Rodriguez et al.,^10^ que apontam para uma alta incidência de morte não cardíaca, incidência de câncer ou ambas em pacientes tratados com SF contemporâneos em comparação com a ponte de safena cirúrgica ou terapia médica otimizada como comparador (5,5% e 3,8%, p = 0,000018).

Uma discrepância semelhante na mortalidade também foi destacada em vários ensaios e metanálises que compararam stents farmacológicos com balões revestidos com fármacos,^11^ levantando assim preocupações importantes sobre as indicações para implante extenso de stents coronários, especialmente em anatomias particulares, como em vasos pequenos, onde as técnicas sem metal mostraram resultados promissores.

Curiosamente, no registro brasileiro com o Inspiron™ BP-SES 9, apesar da associação significativa entre menor diâmetro vascular e desfechos adversos em análises não ajustadas, o diâmetro vascular médio não emergiu como um preditor independente de eventos cardiovasculares adversos maiores (MACE) após o ajuste por regressão de Cox multivariável.

De fato, o perfil mais fino desse stent mais recente poderia ter oferecido uma área luminal maior e melhorado o fluxo sanguíneo. Notavelmente, não foram fornecidos dados sobre a taxa real de vasos muito pequenos (<2,5 mm), e nem foram consideradas outras características anatômicas importantes, como lesões de bifurcação, manejo do cálcio e o uso de imagens intracoronárias, que comprovadamente melhoram os resultados da ICP, especialmente em casos selecionados.

Como esperado, no estudo de Soccol et al.,^9^ foi encontrada uma interação significativa de acordo com a apresentação aguda versus estável, confirmando os melhores resultados já estabelecidos em pacientes com síndrome coronariana crônica.

De fato, o maior aumento de MACE neste estudo ocorreu após 12 meses, ou seja, após a DAPT, sugerindo que uma terapia antitrombótica dupla prolongada nesses pacientes de alto risco poderia ter oferecido benefícios potenciais.

Contudo, no presente registo, a única indicação do protocolo foi uma duração mínima de DAPT de 6 meses, não tendo sido recolhidos detalhes sobre a DAPT.

No entanto, a introdução de uma quarta geração de SF altamente sofisticados e sem polímeros reduziu ainda mais, mas não eliminou, o risco de eventos cardiovasculares relacionados ao stent, muitas vezes exigindo um equilíbrio entre a redução do sangramento com regimes de DAPT mais curtos e um potencial aumento na revascularização do vaso-alvo.^12^

Assim, mesmo confiando na presença, em nossos laboratórios de cateterismo, de SF contemporâneos extremamente seguros e eficazes, que representam o melhor da tecnologia que podemos oferecer aos nossos pacientes, não podemos ignorar os riscos residuais relacionados ao seu implante. Portanto, uma abordagem personalizada deve ser priorizada, considerando opções alternativas sem metal para ICP ou não ICP, em favor da revascularização cirúrgica ou da terapia medicamentosa otimizada, de acordo com um equilíbrio individualizado entre a anatomia coronária, o perfil de risco clínico e a preferência do paciente.

## References

[1] Brami P, Fischer Q, Pham V, Seret G, Varenne O, Picard F (2023). Evolution of Coronary Stent Platforms: A Brief Overview of Currently Used Drug-Eluting Stents. J Clin Med.

[2] Verdoia M, Kedhi E, Suryapranata H, Galasso G, Dudek D, De Luca G (2020). Polymer-Free vs. Polymer-Coated Drug-Eluting Stents for the Treatment of Coronary Artery Disease: A Meta-Analysis of 16 Randomized Trials. Cardiovasc Revasc Med.

[3] Piccolo R, Pilgrim T (2017). The Impact of Thin-Strut, Biodegradable Polymer Stent Designs. Card Interv Today.

[4] Kalra A, Rehman H, Khera S, Thyagarajan B, Bhatt DL, Kleiman NS (2017). New-Generation Coronary Stents: Current Data and Future Directions. Curr Atheroscler Rep.

[5] Baptista SB (2021). The Third Generation of Drug-Eluting Stents: Reassuring Data While We Wait for the Next One. Rev Port Cardiol.

[6] Kobayashi T, Sotomi Y, Suzuki S, Suwannasom P, Nakatani S, Morino Y (2020). Five-Year Clinical Efficacy and Safety of Contemporary Thin-Strut Biodegradable Polymer versus Durable Polymer Drug-Eluting Stents: A Systematic Review and Meta-Analysis of 9 Randomized Controlled Trials. Cardiovasc Interv Ther.

[7] Bangalore S, Toklu B, Amoroso N, Fusaro M, Kumar S, Hannan EL (2013). Bare Metal Stents, Durable Polymer Drug Eluting Stents, and Biodegradable Polymer Drug Eluting Stents for Coronary Artery Disease: Mixed Treatment Comparison Meta-Analysis. BMJ.

[8] Prado GFA, Abizaid AAC, Meireles GC, Sarmento-Leite R, Prudente M, Cantarelli M (2021). Comparative Clinical Performance of Two Types of Drug-Eluting Stents with Abluminal Biodegradable Polymer Coating: Five-Year Results of the DESTINY Randomized Trial. Rev Port Cardiol.

[9] Soccol RC, Ribeiro HB, Lasevitch R, Pellegrini D, Silva EER, Mattos CAS (2026). Desfechos em 2 Anos no Mundo Real do Stent Inspiron™ com Eluição de Sirolimo e Polímero Biodegradável. Arq Bras Cardiol.

[10] Rodriguez AE, Fernandez-Pereira C, Mieres JR, Rodriguez-Granillo AM (2023). High Non-Cardiac Death Incidence Should Be a Limitation of Drug-Eluting Stents Implantation? Insights from Recent Randomized Data. Diagnostics.

[11] Verdoia M, Nardin M, Rognoni A, Cortese B (2024). Drug-Coated Balloons in High-Risk Patients and Diabetes Mellitus: A Meta-Analysis of 10 Studies. Catheter Cardiovasc Interv.

[12] Ellert-Gregersen J, Jensen LO, Jakobsen L, Freeman PM, Eftekhari A, Maeng M (2022). Polymer-Free Biolimus-Coated Stents versus Ultrathin-Strut Biodegradable Polymer Sirolimus-Eluting Stents: Two-Year Outcomes of the Randomised SORT OUT IX Trial. EuroIntervention.

