# Supplementary material for: Stents Farmacológicos de Polímero Bioabsorvivel: Uma Presença Sólida nos Laboratórios de Cateterismo, mas será Suficiente?
Source: Arq Bras Cardiol. 2026 Nov 4;123(9):e20260668. [Article in Portuguese] doi: 10.36660/abc.20260668 (PMC13754396; doi:10.36660/abc.20260668)
Supplement: Translation

# Translation: Bioresorbable Polymer Drug-Eluting Stents: A Solid Presence in Cath-Labs, but Enough?

**DOI:** 10.36660/abc.20260668i

**Authors:** Monica Verdoia, Maria Antonieta Albanez, Andrea Rognoni

**Affiliations:** 1 Ponderano Italy Nuovo Ospedale degli Infermi, Ponderano – Italy; 2 Recife PE Brazil Real Hospital Português, Recife, PE – Brazil

**Keywords:** Bioresorbable Polymer Drug Eluting Stents, Restenosis, Clinical Outcomes

Several generations of drug-eluting stents (DES) have followed, trying to reduce the invasiveness of metal struts and device-related complications. Yet, despite the achievement of very low rates of stent thrombosis (<1%) and restenosis (<5%), these events still impact the outcomes of patients with coronary artery disease, especially in more complex anatomical or clinical subsets.^1,2^

The progressive reduction of metal strut thickness, with the development of new materials and stent design, has allowed to improve trackability and prevented the excessive caging of the vessel, being more respectful of vasomotion and side branches. In addition, diminishing the traumatic and inflammatory stimulus with thinner struts could account for fewer restenotic events.^3^

Indeed, current generations of DES, ranging from 60 to 100μm, have probably reached the minimum strut resolution, paying, conversely, the fee of reduced radial force and potential fractures, foreshortening and under-expansion, especially in calcific lesions.^3^

Targeting restenosis with local delivery of immune- system modulators, eluted on the abluminal side of the stent, has certainly represented the turning point of DES, with the development of the "-*limus*" family, combining the antiproliferative and anti-inflammatory effects with biocompatibility properties. However, the first polymers, used to modulate the progressive release of these drugs to the vessel wall, turned out to be themselves pro-inflammatory and thrombogenic.^1,4^

The third generation of DES has tried to overcome this limitation by introducing bioresorbable (BP) polymers, such as Polylactic acid (PLA) and Poly(lactic-co-glycolic acid (PLGA), which completely disappear by the time the drug elution is completed, generally within 3 to 12 months after implantation.

Multiple 3^rd^ generation DES, featuring minor differences, as shown in Table 1, have been developed, showing consistent non- inferiority against permanent polymer DES in their validation trials.^2,5^

**Table 1:** Current bioresorbable polymer thin-strut drug-eluting stents (DES)

DES Brand	Struts material	Struts thickness	Eluted drug	Polymer type	Polymer duration	Short DAPT validation	Clinical endpoints assessment	MACE	TLR	ST
Inspiron ™	Chromium-cobalt	75 μm	Sirolimus	PLA + PLGA	6-9 months	X	24 months	6.7%	0.9%	0.6%
Synergy ™ XD	Platinum-cobalt	74 μm	Everolimus	PLGA	3-4 months	√	12 months	-	2.6%	0.4%
Orsiro ™	Chromium-cobalt	61 μm	Sirolimus	PLLA	12-24 months	√	60 months	-	6.3%	0.7%
Biomatrix Alpha ™	Stainless steel	112 μm	Biolimus A9	PA	9 months	X	60 months	15.7%	9.4% *	2.2%
DESyne BD/BDS ™	Chromium-cobalt	81 μm	Novolimus/Sirolimus+ rivaroxaban+Argatroban	PLLA	6-9 months	X	24 months	-	2.1-4.2%	0-1%
Biomime ™	Chromium-cobalt	65 μm	Sirolimus	PLLA+PLGA	1-2 months	X	24 months	7.7%	5.95%	1.19%
Mistent ™	Chromium-cobalt	59 μm	Sirolimus	PLLA+PLGA	1.5-2 months	X	60 months	15.1%	3.5%	0%
Firehawk ™	Chromium-cobalt	86 μm	Sirolimus	PA	6-9 months	√	60 months	-	7.6%	2.6%
Combo ™	Stainless steel	100 μm	Sirolimus + CD34^+^ luminal coating	PA	3 months	√	12 months	-	2.5%	0.7%
Supraflex Cruz ™	Chromium-cobalt	60 μm	Sirolimus	PLLA+PLCL+PVP	9-12 months	√	36 months	-	6.3%	1%
Ultimaster ™	Chromium-cobalt	80 μm	Sirolimus	PLCL	3-4 months	√	12 months	-	1.7%	0.7%
Yukon Choice ™	Stainless steel	79 μm	Sirolimus	PA	2-3 months	√	120 months	47.7%	20.3%	2.5%
Nobori ™	Stainless steel	120 μm	Biolimus A9	PA	9.12 months	X	60 months	19.9%	6.3%	0%

* for target vessel revascularization.

PLA: polylactic acid; PLGA: poly lactic-co-glycolic acid; PA: poliammide; PLCL: Poly L-lactide-co-ε-caprolactone; PVP: Polyvinylpyrrolidone; DAPT: dual antiplatelet therapy; MACE: major adverse cardiovascular events; TLR: target lesion revascularization; ST: stent thrombosis.

However, as confirmed by recent meta- analyses,^6,7^ no significant reduction in stent-related events was observed with the newest DES, displaying low but still relevant rates of lesion failure (TLF) and clinical events (7-10% at 1 year and average 15% at 5 years).

The Inspiron™ stent is an ultrathin (75μm) BP-sirolimus eluting stent (Scitech, Aparecida de Goiania, Brazil) currently representing one among the few 3^rd^ generation DES to be tested in its first-in-man study against another BP-DES.^8^ In fact, the DESTINY trial randomized 2:1 170 patients with 194 lesions, to receive Inspiron™ BP-SES vs Biomatrix BP-BES, showing comparable rates of combined major adverse cardiac events (MACE, cardiac death, myocardial infarction, or target lesion revascularization) at 5 years and no case of stent thrombosis.

In the current paper by Soccol et al.^9^, the Inspiron™ BP- SES was used in a much larger real-life cohort of 2,803 patients treated in 25 centers in Brazil. They included complex lesions, mostly type B2 or C and requiring more than one stent, with 5% ISR, and high- risk subsets of patients (around 50% with acute coronary syndrome and over 30% with diabetes mellitus). Despite this, the rate of MACE at 2 years was extremely low, only 6.7%, while TLF and stent thrombosis resulted in line with previous literature. Of note, the inclusion of cardiac death, rather than all-cause mortality in the MACE definition, could have partially accounted for the lower rate of events in this study.

In effect, overall death occurred in about 5% of the patients, in accordance with other large-scale registries and RCT, and with the conclusions of the pooled analysis by Rodriguez et al.^10^ pointing at a high incidence of non-cardiac death, cancer incidence, or both in patients treated with contemporary DES as compared to the surgical bypass or optimal medical therapy as a comparator (5.5% and 3.8%, p = 0.000018).

A similar discrepancy in mortality has also been underlined in several trials and meta-analyses comparing DES with drug-coated balloons.^11^ thus raising important concerns on the indications for extensive coronary stenting, especially in particular anatomies, such as in small vessels, where metal-free techniques have shown promising results.

Interestingly, in the Brazilian registry with the Inspiron™BP-SES^9^, despite the significant association between smaller vessel diameter and adverse outcomes in unadjusted analyses, mean vessel diameter did not emerge as an independent predictor of MACE after multivariable Cox regression adjustment.

Indeed, the thinner profile of this newer stent could have offered a larger luminal area and improved flow. Notably, no data about the actual rate of very small (<2.5 mm) vessels were provided, and neither other important anatomical features were considered, such as bifurcation lesions, management of calcium, and the use of intracoronary imaging, which has been shown to improve the outcomes of PCI especially in selected cases.

As expected, in the study by Soccol et al.,^9^ a significant interaction was found according to acute vs stable presentation, confirming the established improved outcomes in patients with chronic coronary syndrome.

Indeed, the larger rise of MACE in this study occurred after 12 months, thus after DAPT discontinuation, suggesting that a prolonged dual antithrombotic therapy in these high-risk patients could have offered potential benefits.

However, in the present registry, the only protocol indication was a minimum DAPT duration of 6 months, whilst details on DAPT were not collected.

Nevertheless, the introduction of a 4th generation of highly engineered polymer- free DES has further tapered but not abolished the risk of stent-related CV events, often requiring balancing between the reduction of bleeding with shorter DAPT regimens and a potential increase in target vessel revascularization.^12^

Thus, as we rest in the certainties of the presence in our cath-labs of extremely safe and effective contemporary DES, representing the best in technology we can offer to our patients, we cannot scotomize the residual risks related to their implantation. Therefore, a tailored approach should be preferred, considering alternative metal-free options for PCI or no PCI, in favor of surgical revascularization or optimal medical therapy, according to an individualized balancing between coronary anatomy, clinical risk profile and patients’ preference.
